# Quality, Reliability and Accuracy of Hyperthyroidism‐Related Content on Social Media Platform TikTok

**DOI:** 10.1002/edm2.70105

**Published:** 2025-09-10

**Authors:** Aayush Shah, Raika Bourmand, Freddy Albaladejo, Karthik Jarugula, Sofia Olsson, Zainab Farzal, Viraj Shah

**Affiliations:** ^1^ University of Florida Gainesville Florida USA; ^2^ Texas Christian University Anne Burnett Marion School of Medicine Fort Worth Texas USA; ^3^ University of Central Florida Orlando Florida USA; ^4^ Department of Otolaryngology ‐ Head and Neck Surgery University of California Davis Health Sacramento California USA; ^5^ Department of Otolaryngology‐Head and Neck Surgery University of Texas Southwestern Medical Center Dallas Texas USA

**Keywords:** digital health information, hyperthyroid, social media, TikTok

## Abstract

**Objective(s):**

To evaluate the quality, reliability and accuracy of hyperthyroidism‐related content on TikTok using validated assessment tools.

**Methods:**

We systematically searched TikTok for ‘hyperthyroid’ and ‘high thyroid’, analysing 115 videos after exclusions. Two independent researchers assessed videos using the Global Quality Scale (GQS, range 0–5) for overall content quality, the modified DISCERN (mDISCERN, range 0–5) for reliability and the Accuracy in Digital Information (ANDI, range 0–4) tool for factual correctness. We categorised creator credentials and content purpose, performing statistical analyses to examine associations with video quality and engagement.

**Results:**

Of the 115 videos analysed, the mean ANDI score was 3.15/4, the mean GQS was 2.72/5, and the mean mDISCERN score was 2.47/5. Educational content (98.3%) demonstrated higher GQS (*p* = 0.019) and mDISCERN (*p* = 0.040) scores than non‐educational content. Conversely, anecdotal content (35.7%) was associated with significantly lower GQS (*p* = 0.002) and mDISCERN (*p* < 0.001) scores. Healthcare professionals (HCPs, 37.4% of creators) produced videos with higher ANDI (*p* = 0.015), GQS (*p* < 0.001) and mDISCERN (*p* < 0.001) scores than non‐HCPs. Notably, physician‐created videos garnered higher engagement across all metrics (*p* < 0.05).

**Conclusions:**

While some TikTok content on hyperthyroidism is of high quality, particularly from healthcare professionals, the platform is dominated by lower quality content from non‐experts. This underscores the need for increased engagement from healthcare professionals on social media to improve the accuracy and reliability of health information available to the public.

## Graphical abstract

TikTok is a major source of hyperthyroidism information, but most content is anecdotal and produced by non‐experts. Physician‐created videos are more accurate, reliable and higher quality, yet underrepresented. Greater professional engagement is needed to ensure trustworthy health information on social media.

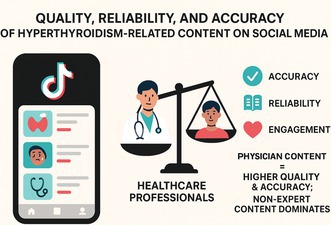

## 1 Introduction

In recent years, social media has expanded as a popular source of health information among the general population [1]. Platforms like TikTok [2], now ranking fifth worldwide in terms of monthly active users, have become especially common for sharing and consuming medical content [3]. A recent study reported that 92.4% of respondents had unintentionally received health information on TikTok [4]. While the accessibility of social media offers opportunities for health education, there are concerns regarding the accuracy of information being shared. Many users believe that inaccurate or misleading health content is prevalent on the platform [4, 5].

This issue is particularly concerning when health information relates to medical conditions that require nuanced understanding and timely management. One such condition is hyperthyroidism. Hyperthyroidism is an endocrine disorder defined by low thyroid stimulating hormone (TSH) with excessive production of thyroid hormones, including triiodothyronine (T3) and/or free thyroxine (T4) [6]. This condition affects 0.2%–1.3% of individuals globally and can vary in its symptoms and aetiology [7]. Hyperthyroidism may present with symptoms overlapping with other conditions leading to misdiagnosis. For example, it is often mistaken as anxiety or panic disorder due to its ability to cause tremors or cardiovascular complications [8, 9]. Due to this varied presentation, it is critical that patients with complaints within and beyond the specialty of otolaryngology have access to trustworthy information about the condition. TikTok features content from both healthcare professionals and non‐professionals, which can lead to a mix of accurate information and content that may be oversimplified or misleading [2].

This study aims to offer a comprehensive evaluation of the quality, reliability and accuracy of TikTok content related to hyperthyroidism using a series of validated assessment tools. By doing so, it will help inform both content creators and healthcare professionals on the landscape of endocrine health communication on social media and better support efforts to improve public health literacy in the digital age.

## 2 Materials and Methods

From December 9, 2024 to December 11, 2024, two independent researchers systematically queried the terms ‘hyperthyroid’ and ‘high thyroid’ on TikTok. These terms were selected to represent both medical and colloquial interpretations of the condition with the goal of viewing videos from creators with various backgrounds and understanding. Additionally, the two terms were chosen to encompass all possible causes of hyperthyroidism. Specific conditions, such as Graves' disease, were excluded to prevent disease‐based bias in our results as they may present with unique content profiles.

To prevent algorithmic personalisation, newly created TikTok accounts with no prior history were used. Each researcher independently recorded the first 70 videos yielded by each search term using the platform's default algorithm ranking. This limit was established based on preliminary observations indicating decreased content relevance beyond this point. After removing duplicates from the initial collection, 147 unique videos remained, comprising the data set for analysis. An additional 32 videos were excluded for being unrelated to hyperthyroidism (*n* = 12) or lacking sufficient claims to be rated (*n* = 20), resulting in a final dataset of 115 videos. This data collection process was based on previously published literature [10, 11, 12, 13, 14]. A custom Python script was developed to extract video metadata, including engagement metrics (view, like, share and comment counts), video duration, creation timestamp, creator characteristics and indicators of paid promotion.

Multiple validated assessment tools were used to evaluate the quality of health information presented in the data set. The Global Quality Scale (GQS, range 1–5) [15] provides a general measure of the overall quality and flow of information, taking into consideration clarity and comprehensiveness. The modified DISCERN (mDISCERN, range 1–5) [15] tool is used to assess the reliability through factors like source citation and clarity of treatment options. The Accuracy in Digital Information (ANDI, range 0–4) score is used to determine actual factual accuracy of content in comparison to medical guidelines and evidence. Information was described as accurate based on the current hyperthyroidism guidelines including initial evaluation or management of conditions like Grave's disease, thyrotoxicosis, goitres or adenomas [16]. These tools have been readily used by similar studies to date, making them well‐suited for systematically assessing TikTok content validity [17, 18, 19]. Each video was evaluated by two independent researchers using standardised instruments. Mean scores from both raters were used for final ratings. Inter‐rater reliability for ANDI, GQS and mDISCERN scores was assessed using quadratic‐weighted Cohen's Kappa to account for the ordinal nature of the scales.

To ensure consistent categorisation of video content purpose, a standardised codebook was developed. This codebook, available as Table S1, was created specifically for classifying videos as educational, anecdotal or entertainment‐focused. It provided clear operational definitions, inclusion and exclusion criteria, and illustrative examples for each of these three content purpose types. The primary aim of this codebook was to ensure that all members of the research team had a shared understanding of these categories and could apply them uniformly during video analysis. For this reason, the definitions and examples within the codebook were collaboratively developed and reviewed by the authors. Any ambiguities in the interpretation of these categories were discussed and clarified by the team until a consensus on the final version was reached prior to its use. Using this established codebook, videos were then manually coded for their primary content purpose or purposes. A single video could be assigned multiple purpose codes if its content met the criteria for more than one of these defined categories.

Creator credentials were classified as healthcare professionals (HCPs) and non‐healthcare professionals (non‐HCPs). HCPs were strictly defined as individuals holding specific, verifiable qualifications, such as physicians, nurse practitioners and physician assistants. Other HCPs were defined as a subcategory of HCPs that included nurses, physician assistants and other allied health professionals who were not physicians with an allopathic (MD) or osteopathic (DO) medical degree. Identification of clinical content relating to specific hyperthyroidism‐related information involved a two‐stage process. Initially, coders manually recorded all pertinent symptoms and treatments mentioned within each video. These manually compiled lists were programmatically consolidated, and duplicates were removed using a custom algorithm in the Python software to derive accurate frequency counts for analysis.

Statistical analyses were performed using Python 3.11.10 with libraries including statsmodels, scipy, pandas and numpy. Descriptive statistics (count, mean, standard deviation, median, range, frequencies and proportions) were calculated for all variables. Mann–Whitney U tests, with Cohen's *d* for effect size, were used to compare quality metrics based on content purpose (e.g., educational vs. non‐educational) and to compare quality metrics and video duration between creator groups (e.g., HCP vs. non‐HCP; MD/DO vs. non‐HCP). *P* values for these group comparisons were adjusted using the Benjamini‐Hochberg false discovery rate method. Multiple linear regression analyses were conducted to examine predictors of log‐transformed engagement metrics, with predictors including quality scores, video duration, creator status (MD/DO) and content purpose, along with relevant interaction terms. Pearson correlation coefficients assessed linear relationships between quality scores, log‐transformed engagement metrics and video duration. Spearman rank correlation coefficients assessed monotonic relationships between quality scores and author profile metrics. Statistical significance was defined as a *p* value ≤ 0.05 for all analyses.

## 3 Results

### 3.1 Video Characteristics and Creator Demographics

The final analysis included 115 unique TikTok videos addressing hyperthyroidism. Descriptive statistics for video quality scores, engagement metrics, content purpose and creator demographics are presented in Table 1. On accuracy metrics as described by ANDI, videos scored a mean of 3.15 out of 4 (median = 4.00) indicating generally high accuracy. Video quality as described by the GQS scored a mean of 2.72 out of 5 (median = 3.00) indicating low to moderate quality. Reliability as described by mDISCERN scored 2.47 out of 5 (median = 2.50) indicating low reliability.

**TABLE 1:** Descriptive statistics of video characteristics and creator demographics (blank indicates data unavailable).

Variable	Mean (SD)	Median (Min–Max)	*N* (%)
Video quality scores			
ANDI score (0–4)	3.15 (1.30)	4.00 (0.00–4.00)	
GQS score (1–5)	2.72 (0.96)	3.00 (1.00–5.00)	
mDISCERN score (1–5)	2.47 (1.01)	2.50 (1.00–5.00)	
Video metrics			
View count	644,044 (1,937,340)	48,300 (616–13,800,000)	
Like count	12,725 (38,146)	1006 (0–265,300)	
Share count	746 (1823)	72 (0–10,800)	
Comment count	185 (359)	59 (0–2157)	
Video duration (s)	73.66 (88.50)	49.00 (5.00–577.00)	
Content purpose			
Educational			113 (98.3%)
Anecdotal			41 (35.7%)
Paid promotion			7 (6.1%)
Entertainment			1 (0.9%)
Creator type			
Non‐healthcare professionals			72 (62.6%)
Physicians (MD/DO)			25 (21.7%)
Other healthcare professionals			18 (15.7%)
Creator gender			
Female			92 (80.0%)
Male			21 (18.3%)
Neither			2 (1.7%)

*Note:* *N* = 115 videos analysed. Percentages for content purpose, creator type and creator sex (videos by) are based on *N* = 115 videos.

Video engagement was highly variable with a mean (SD) view count of 644,044 (1,937,340), like count of 12,725 (38,146), share count of 746 (1823), and comment count of 185 (359). The average video duration was 73.66 (88.50) s. Most videos were categorised as having an educational purpose (98.3%), with a notable portion also containing anecdotal content (35.7%). Paid promotions accounted for 6.1% of videos. HCPs created 37.4% of the videos, with physicians authoring 21.7% of all videos. Non‐HCPs constituted the largest creator group (62.6%), followed by physicians (21.7%) and Other HCPs (15.7%). The majority of creators were female (80.0%).

### 3.2 Inter‐Rater Reliability

Inter‐rater reliability for the quality assessment tools was almost perfect, with quadratic‐weighted Cohen's kappa values of 0.877 (95% CI 0.743–1.010) for ANDI, 0.866 (95% CI 0.676–1.056) for GQS and 0.922 (95% CI 0.747–1.097) for mDISCERN. This suggests minimal disagreement between raters and more reliable results.

### 3.3 Association Between Content Purpose and Video Quality

Content purpose was significantly associated with video quality, accuracy and reliability scores. Educational videos demonstrated higher GQS indicating good video quality and flow (*p* = 0.019) and higher mDISCERN scores indicating higher reliability (*p* = 0.040) compared to non‐educational videos. Conversely, anecdotal content was linked to significantly lower GQS (*p* = 0.002) and mDISCERN scores (*p* < 0.001). Paid promotion content exhibited significantly lower ANDI scores (*p* < 0.001) indicating more inaccurate information being provided. Other comparisons of quality by content purpose, such as ANDI scores for educational or anecdotal videos, were not statistically significant (*p* > 0.05).

### 3.4 Association Between Creator Type and Video Quality

**FIGURE 1:**
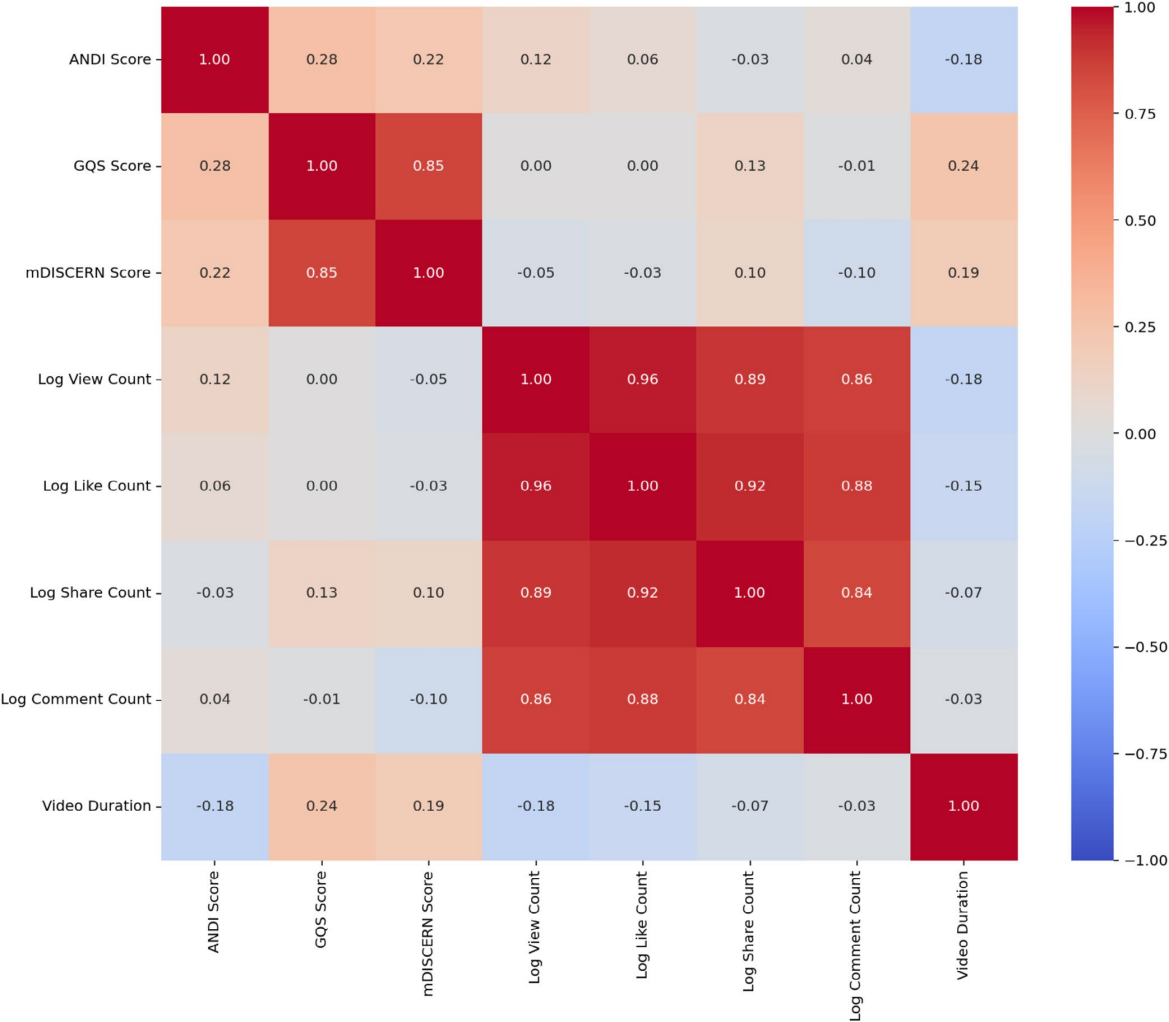
(A) Mean ANDI score by creator type (B) Mean GQS by creator type (C) Mean mDISCERN by creator type.

Creator type was significantly associated with video quality, accuracy, reliability scores and duration, after Benjamini‐Hochberg correction for multiple comparisons. Videos by HCPs achieved higher ANDI (*p* = 0.015), GQS (*p* < 0.001) and mDISCERN scores (*p* < 0.001) than those by non‐HCPs. Specifically, MD/DO creators' videos had higher ANDI (*p* = 0.011) and mDISCERN scores (*p* = 0.015). Compared to Other HCPs, videos by MD/DOs had lower GQS (*p* = 0.015) indicating lower overall video quality and flow despite higher scores in other measures. Videos from Other HCPs demonstrated higher GQS (*p* < 0.001) and mDISCERN scores (*p* < 0.001) than those from non‐HCPs, indicating better overall quality and higher reliability (Table 2 and Figure 1).

**TABLE 2:** Comparison of video quality scores by creator type (bolded *p* value indicates significance).

Metric	Group 1 mean	Group 2 mean	*p*
HCP vs. non‐HCP (Group 1 vs. Group 2)			
ANDI	3.55	2.92	**0.020**
GQS	3.16	2.46	**0.001**
mDISCERN	2.95	2.19	**< 0.001**
MD/DO vs. non‐HCP (Group 1 vs. Group2)			
ANDI	3.74	2.916667	**0.011**
GQS	2.86	2.458333	0.070
mDISCERN	2.76	2.1875	**0.015**
MD/DO vs. Other HCP (Group 1 vs. Group2)			
ANDI	3.74	3.277778	0.168
GQS	2.86	3.583333	**0.015**
mDISCERN	2.76	3.222222	0.058
Other HCP vs. Non‐HCP (Group 1 vs. Group2)			
ANDI	3.277778	2.916667	0.291
GQS	3.583333	2.458333	**< 0.001**
mDISCERN	3.222222	2.1875	**< 0.001**

*Note:* Mann–Whitney *U* test was used for comparisons. Corrected *p* values are reported. Other HCP refers to HCP's that are not MD/DO.

Abbreviation: HCP, healthcare professional.

### 3.5 Predictors of Video Engagement

Multiple linear regression indicated that being a MD/DO creator was a significant positive predictor for log‐transformed video view count (*β* = 2.188, *p* = 0.008), like count (*β* = 1.923, *p* = 0.025), share count (*β* = 1.805, *p* = 0.029) and comment count (*β* = 1.188, *p* = 0.044). These models explained 14.7%–19.4% of the variance in engagement (*R* ^2^ values 0.194, 0.147, 0.153 and 0.174, respectively). Video quality scores (ANDI, GQS, mDISCERN), duration, other content purpose flags and interaction terms did not significantly predict engagement (*p* > 0.05).

### 3.6 Correlations Between Metrics

**FIGURE 2:**
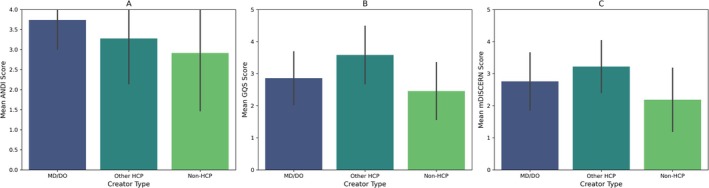
Pearson correlation matrix of video quality scores (ANDI Score, GQS Score, mDISCERN Score), log‐transformed engagement metrics, and video duration. Values within the heatmap cells represent Pearson correlation coefficients (*r*). The colour scale indicates the strength and direction of the correlations.

GQS and mDISCERN scores were strongly positively correlated (*ρ* = 0.830) with each other. Quality, reliability and accuracy scores demonstrated weak correlations with author profile metrics, such as mDISCERN versus follower count (*ρ* = 0.238). Log‐transformed engagement metrics were highly intercorrelated (log view count vs. log like count, *r* = 0.957). Consistent with regression analyses, correlations between the three scores and log‐transformed engagement were weak (ANDI vs. log view count, *r* = 0.125). Video duration had a weak negative correlation with log‐transformed view count (*r* = −0.182) (Figure 2).

### 3.7 Content Analysis of Symptoms and Treatments

**FIGURE 3:**
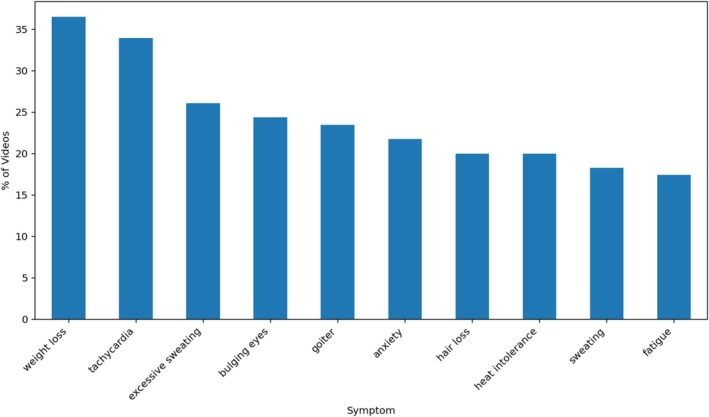
The top 10 most mentioned symptoms within the videos analysed.

**FIGURE 4:**
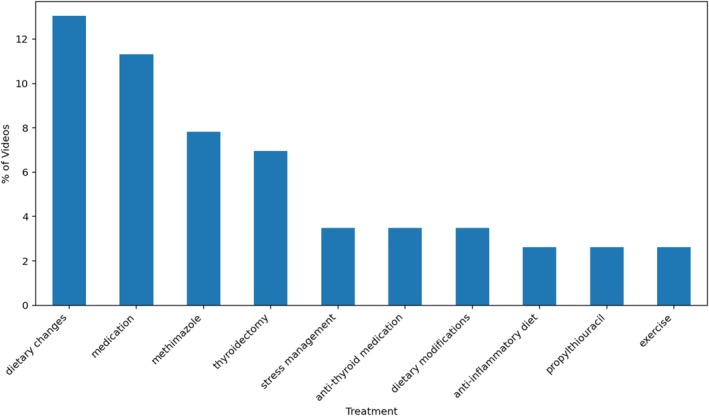
The top 10 most mentioned treatments within the videos analysed.

The most frequently mentioned symptoms of hyperthyroidism included weight loss (36.5% of videos), tachycardia (33.9%), excessive sweating (26.1%), bulging eyes (24.3%) and goitre (23.5%). Commonly cited treatments were dietary changes (13.0%), unspecified medication (11.3%), methimazole (7.8%) and thyroidectomy (7.0%) (Figures 3, 4).

## 4 Discussion

The present study is the first of its kind to provide a detailed analysis of TikTok videos addressing hyperthyroidism, highlighting key trends in the quality of content, demographics of creators and the engagement of the viewing audience.

Overall, we found that the average ANDI, GQS and mDISCERN scores were all lower than the median value. This reveals that while some content did meet basic standards of reliability and trustworthiness, there is significant room for improvement. Educational content was found to be the most prevalent category, making up nearly all videos and was associated with significantly higher GQS and mDISCERN scores, a finding supported by previous literature [20, 21]. Educational videos likely scored higher because they used evidence‐based language, presented clear learning objectives and avoided the bias of anecdotal accounts. About one third of the videos were anecdotal, and these consistently received lower quality scores. Similar findings have been reported in other TikTok studies showing that patient journey or anecdotal content tends to score lower than educational posts created by professionals, including those on thyroidectomy, genitourinary cancers and acne. Anecdotal material may be more emotionally engaging, yet it often lacks the balance or references needed for high‐quality education. This resonates with a well‐documented challenge of balance in social media communication, which is that of relatability and reliability, with videos often steering toward one or the other [22]. Additionally, it may be preferred by content creators to share personal stories or simplified messaging to increase viewer engagement, even if this is at the expense of informational depth.

The monetisation structure of TikTok, which rewards creators for higher views and engagement, further incentivises this trade‐off [23]. The willingness of creators to attract engagement at the expense of misinformation has previously been studied on the social media platform, Twitter [24]. In a study of 126,000 stories that were shared more than 4.5 million times, it was found that the false information received much more attention in a shorter amount of time than accurate information across science and other categories [25]. This can be further applied to the present study where paid promotions accounted for 6.1% of the videos and performed poorly on the ANDI assessment, as previously found by other investigations [26]. Ultimately, it is important to consider whether funding creators based on solely engagement inhibits their willingness to share reliable information and the role of platforms like TikTok in enabling such creators to do so.

In our study, physicians achieved significantly higher ANDI and mDISCERN scores than non‐HCPs, a pattern also observed in other endocrine‐related TikTok studies focused on thyroidectomy and thyroid cancer, as well as in hypertension [27, 28, 29, 30]. Interestingly, the videos by physicians were found to be shorter in duration than other groups, yet still achieved higher engagement scores, suggesting that high‐quality information does not necessarily depend on lengthy communication. Moreover, it suggests that viewers may inherently trust physicians more, clear medical authority being a powerful differentiator, supported by previous literature demonstrating physicians having the greatest viewer credibility on TikTok [31]. The concept of effective communication of HCPs on TikTok is consistent across other medical conditions, such as human papillomavirus [32], genitourinary cancers [33] and lasik surgery [34]. Despite this, physician content was outperformed in terms of GQS by other healthcare professionals (e.g., nurse practitioners, physician assistants, pharmacists), drawing attention to the potentially more patient‐centric or concise communication style among such providers. This is consistent with other research showing that high‐quality health content does not necessarily drive clicks or shares [35]. Evidently, the algorithm of TikTok prioritises watch time, entertainment value and trend participation rather than accuracy. This disconnect highlights a challenge in digital health; the qualities that promote engagement are often not connected to those that determine accuracy. Therefore, health communication must include both of such things in order to appeal to both platform algorithms and user expectations.

There are multiple strengths of this study. It is the first to collect information regarding the quality, reliability and accuracy of hyperthyroid‐related TikTok content. The collection of videos from the user interface provided a more accurate reality of the videos which audiences within the United States might encounter. Unlike several prior TikTok studies that sampled only the ‘top liked’ or hashtag‐specific videos, our approach captured consecutively surfaced videos from two general search terms on new accounts, reducing popularity bias and approximating how average users encounter information [30, 36]. Data collection and thematic analysis were performed by two independent researchers and yielded strong inter‐rater reliability, minimising bias and misinterpretation. While most content audits rely on DISCERN and GQS alone, we also incorporated the ANDI tool to directly assess guideline‐based factual accuracy. In addition, our engagement analyses used multivariable regression to account for creator type, video duration and interaction terms, whereas most earlier studies relied on bivariate correlations.

However, the study has several limitations. First, the cross‐sectional design captures only a single snapshot in time and does not account for changes in TikTok's algorithm or user trends; thus, engagement metrics are dynamic and may evolve after data collection. Second, although new accounts with no watch history were used to limit personalisation, TikTok recommendations can still be influenced by geography, device or other algorithmic factors. Because all searches were conducted in the United States during a 3‐day window (December 9–11, 2024), the results may not reflect what users in other regions, languages or time periods would encounter. Third, while interrater reliability was excellent, quality assessments inherently retain some subjectivity, which may introduce bias. Finally, only English‐language content was analysed, limiting the generalisability of these findings to global audiences.

## 5 Conclusion

TikTok offers a promising and precarious opportunity for health information about hyperthyroidism. While educational content, specifically from physicians and other healthcare professionals, demonstrates higher quality and engagement, the overall landscape is actively dominated by non‐expert voices and anecdotal narratives. This emphasises a critical need within the otolaryngology community to actively engage in digital spaces to counter misinformation and ultimately reshape the delivery and consumption of credible information. It is critical to bridge the gap between reliable medical information and audience engagement, which demands new approaches to health communication, algorithmic collaboration and a cultural shift in the perception of authority and reward in digital spaces.

## Author Contributions

**Aayush Shah:** writing of original manuscript, data collection and analysis, review and editing. **Raika Bourmand:** writing of original manuscript, review and editing. **Freddy Albaladejo:** writing of original manuscript, data collection, review and editing. **Karthik Jarugula:** data collection. **Sofia Olsson:** project conception, data collection, review and editing. **Zainab Farzal:** project supervision, review and editing. **Viraj Shah:** project supervision, data collection, review and editing.

## Conflicts of Interest

The authors decalare no conflicts of interest.

## Supporting information


**Table S1:** edm270105‐sup‐0001‐TableS1.docx.

## Data Availability

The data that support the findings of this study are available from the corresponding author upon reasonable request.
