# Supplementary material for: Quality, Reliability and Accuracy of Hyperthyroidism‐Related Content on Social Media Platform TikTok
Source: Endocrinol Diabetes Metab. 2025 Sep 10;8(5):e70105. doi: 10.1002/edm2.70105 (PMC12422942; doi:10.1002/edm2.70105)
Supplement: Supplementary file 1 — Table S1: edm270105‐sup‐0001‐TableS1.docx. [file EDM2-8-e70105-s001.docx]

**Supplemental Table 1: Coding Definitions for Video Quality Assessment**

| **Code (Content Purpose)** | **Definition** | **Example** |
| --- | --- | --- |
| Educational | Explains information relating to hyperthyroidism, aiming to educate the audience. This includes content that discusses symptoms, treatments, causes, prevention, or management strategies in an informative manner. | A medical professional explains hyperthyroidism: detailing key symptoms (e.g., weight loss, rapid heartbeat), common causes (e.g., Graves' disease), and treatment options (e.g., medication, surgery). |
| Entertainment | Content mentioning hyperthyroid-related topics with the primary goal of entertaining the audience. This can include humorous takes, celebrity stories, or creative narratives where hyperthyroidism is part of the storyline but not the central focus of education or factual dissemination. | An influencer creates a comedic skit exaggerating hyperthyroid symptoms like constantly feeling hot, experiencing hand tremors, or having a racing heart for laughs. |
| Anecdotal | Contains personal stories, experiences, or testimonies about hyperthyroidism, where the primary focus is on sharing individual accounts rather than providing generalized information or aiming for entertainment. | An individual recounts their personal hyperthyroidism story: covering their diagnosis process, initial symptoms (e.g., palpitations, weight loss), emotional impact, and treatment experiences. |
